# Dietary Combined Thyme Meal and *Bacillus subtilis* to Promote Growth Performance, Immune Function, Gene Expression, Antioxidant Defense, and Cecal Microbiota in Growing Rabbits Under Heat Stress Conditions

**DOI:** 10.3390/vetsci13020204

**Published:** 2026-02-20

**Authors:** Haifa Ali Alqhtani, Ahmed M. Elbaz, Safaa A. Hegazy, AbdelRahman Y. Abdelhady, Fatmah Ahmed Safhi, Mohamed Marzok, Mohamed Abdo Rizk, Mohammed Al-Rasheed, Mahmoud H. Mohamed, Sherief M. Abdel-Raheem, Ayman E. Taha, Ahmed A. Marwan

**Affiliations:** 1 Department of Biology, College of Science, Princess Nourah bint Abdulrahman University, P.O. Box 84428, Riyadh 11671, Saudi Arabia; haalqhtani@pnu.edu.sa (H.A.A.); faalsafhi@pnu.edu.sa (F.A.S.); 2 Animal and Poultry Nutrition Department, Desert Research Center, Cairo 11753, Egypt; 3 Animal Production Department, Faculty of Agriculture, Ain Shams University, Cairo 11759, Egypt; safaa_abdallah@agr.asu.edu.eg (S.A.H.); ahmed_marwan97@agr.asu.edu.eg (A.A.M.); 4 Poultry Production Department, Faculty of Agriculture, Ain Shams University, Cairo 11759, Egypt; abdelrahman_abdelhady@agr.asu.edu.eg; 5 Department of Clinical Sciences, College of Veterinary Medicine, King Faisal University, P.O. Box 400, Al-Ahsa 31982, Saudi Arabia; mrizk@kfu.edu.sa (M.A.R.); mhmohammad@kfu.edu.sa (M.H.M.); 6 Department of Public Health, College of Veterinary Medicine, King Faisal University, P.O. Box 400, Al-Ahsa 31982, Saudi Arabia; sdiab@kfu.edu.sa (S.M.A.-R.); aetaha@kfu.edu.sa (A.E.T.)

**Keywords:** *B. subtilis*, thyme, growth, immunity, antioxidant, heat stress, gene, growing rabbits

## Abstract

This study investigates the nutritional effects of a thyme meal and *B. subtilis* mixture on growth performance, immune function, antioxidant capacity, gene expression, and gut microbiota in heat-stressed rabbits. One hundred and twenty male New Zealand White rabbits were divided into four dietary treatments (five replicates/group). The rabbits in the first group were fed a basal diet, while the other three groups were fed a basal diet containing *B. subtilis*, thyme meal, and their mixture, respectively. The *B. subtilis* and thyme meal mixture increases the heat-stressed rabbits’ body weight gain and carcass weight, and enhances nutrient digestibility and the feed conversion ratio. Supplementing the CBT mixture improved the lipid profile and liver and kidney function via decreasing plasma triglycerides, cholesterol, LDL, creatinine, urea, and AST levels, while increasing total protein and albumin levels. Furthermore, the CBT mixture enhanced the immune response and oxidative stability by increasing IgA and IgG levels, GPx enzyme activity, and SOD, while decreasing plasma MDA content. Adding the CBT mixture enhanced gut health by reducing pathogens and inflammation, as well as increasing volatile fatty acid levels and the expression of CAT-1, MUC-2, and SGLT-1 genes. The combination of a thyme meal and *B. subtilis* enhanced growth, immune function, antioxidant capacity, gut microbiota modification, and the expression of gut health nutrient absorption-related genes in heat-stressed rabbits.

## Simple Summary

Heat stress is a major obstacle to the sustainability of the rabbit industry. Heat stress causes significant economic losses through its negative impacts on growth performance, intestinal microbiota, gut tissue, immune response, and meat quality, as well as its exposure to oxidative stress. There has been increased interest in nutraceutical supplements to protect rabbits from the harmful effects of heat stress. These nutraceutical supplements include probiotics, organic acids, antioxidants, enzymes, and medicinal plants, due to their diverse properties, which include disease prevention, anti-inflammatory, immunomodulatory, and biotic balance maintenance, and antioxidant activity, thus supporting productive performance. The objective of the current study is to investigate the effect of adding a *B. subtilis* and thyme meal mixture on reducing the impacts of heat stress on the physiological performance and growth of growing rabbits.

## 1. Introduction

Rabbit production plays a vital role in global livestock systems, particularly in developing regions, due to their efficient feed conversion, high reproductive rate, and ability to utilize locally available feed resources [1,2]. Rabbit meat also contributes globally to the supply of high-quality, affordable animal protein and is a significant source of income for smallholder and backyard farmers [3]. In many developing countries, rabbit production supports food security and enhances nutritional value [4], making it a key component of sustainable animal agriculture. Additionally, rabbit meat is characterized by several properties, including its high protein content, low fat content, and unsaturated fatty acids, making it easily digestible and having a distinctive flavor [3,5]. Moreover, genetic improvements in rabbits to achieve higher production performance and higher metabolic rates make them more sensitive to environmental stresses, including high temperatures [6]. During periods of heat stress, rabbits cannot maintain a balance between heat production and heat emission, leading to a series of negative effects on rabbit production [7]. Environmental heat stress causes significant economic losses in the rabbit industry due to its numerous health problems in rabbits, such as oxidative stress, disrupted microbial balance, impaired immune function, endocrine disruption, organ damage, and reproductive disorders, ultimately leading to a decline in production performance [3,8]. Additionally, the weaning period is a critical stage in rabbit breeding, as it is characterized by sudden dietary changes and the incomplete maturation of the digestive system, which leads to the instability of intestinal microbes and incomplete growth of digestive enzyme activity, exposing rabbits to digestive disorders, weakness of the immune system, and intestinal disorders such as bloating and diarrhea, which increase the rates of infection and death [9]. Furthermore, rabbits possess a highly specialized digestive physiology that differs significantly from that of various chicken species [10]. Rabbits rely on intensive microbial fermentation in a well-developed cecum, where synthetic carbohydrates, particularly neutral fiber (NDF) and acid fiber (ADF), are broken down to produce volatile fatty acids (acetate, propionate, and butyrate) [10,11]. Dietary fiber, therefore, plays a pivotal role in shaping the rabbit digestive microbiome and maintaining normal cecal fermentation [12], which may support the use of certain agricultural and/or manufacturing byproducts in rabbit feed. This study investigated the potential of dietary interventions as nutritional strategies to mitigate heat stress and weaning-related challenges in rabbits by the inclusion of selected feed additives [13,14,15,16].

Many byproducts of essential oil production are often overlooked and considered waste. Therefore, it is essential to utilize them due to their numerous properties, thereby reducing environmental waste and promoting resource sustainability [17]. Thyme meal is one such neglected byproduct (representing 65–70% of the fruit) that contains many highly valuable bioactive compounds, such as residual essential oils, phenols, and flavonoids [18,19]. These can be used in animal feed for their therapeutic properties, including antimicrobial and natural antioxidant effects [20]. Additionally, it contains valuable compounds such as protein, carbohydrates, and fiber, as well as biologically active compounds (thymol and carvacrol) and fats that remain from the extraction process [21]. These fats are characterized by their richness in polyunsaturated fatty acids, which supports their use as a nutritional supplement in animal feed [22]. Furthermore, it possesses many potent anti-inflammatory, immunomodulatory, antimicrobial, antioxidant, and digestive-enhancing properties, attributed to its bioactive components [23]. Many reports have also indicated that adding thyme oil or thyme powder to rabbits’ feed enhances the oxidative stability, growth performance, immune response, and intestinal health [21,22,24]. While antimicrobials may benefit by reducing pathogenic bacteria and intestinal inflammation, their overuse can disrupt the balance of fiber-fermenting microbes, alter cecal pH, impair fermentation efficiency, and negatively impact nutrient absorption [25]. Therefore, it is essential to carefully evaluate antimicrobial interventions in rabbits and promote the biological basis of alternatives that support microbial balance to maintain digestive health and efficiency. Therefore, thyme meal was added at concentrations that proved to have beneficial impacts as an antimicrobial and antioxidant [26,27].

Additionally, the use of live beneficial microorganisms (probiotics) as feed additives is widely employed to support animal health and performance [13,28]. Several beneficial microorganisms have been used, including *Bacillus subtilis*, *Lactobacillus acidophilus*, and *Bacillus coagulans*, among others [20,29]. *B. subtilis* possesses several characteristics that support its use as a probiotic source in poultry feed, including its tolerance to harsh conditions (heat and acidity), as well as its anti-inflammatory, immunomodulatory, and antimicrobial properties [29,30]. Many reports have also indicated its positive impact on growth performance through various mechanisms, such as improving metabolism, producing certain enzymes, and modulating amino acid and vitamin B metabolism, thus enhancing feed efficiency [31,32]. Furthermore, it plays a role in promoting intestinal health by modifying the gut microbiota and enhancing the histological structure and junctional integrity [30], thereby boosting disease resistance and overall animal health.

Feed additives have proven beneficial to rabbit performance and health [32]. Also, many reports have indicated the positive effects of aromatic plant supplements and probiotics on disease resistance and mitigating the effects of environmental stress [23,30]. This study utilized a combination of *B. subtilis* and thyme meal supplements to maximize their potential synergistic effects on rabbit performance by alleviating the impacts of weaning problems and heat stress. Therefore, this study evaluated the effects of thyme meal, *B. subtilis*, and their combination on growth performance, redox status, lipid profile, immunity, intestinal integrity, and gene expression.

## 2. Materials and Methods

### 2.1. Experimental Design and Animals

One hundred and twenty male rabbits were provided for the experiment at the Mariout Research Station, affiliated with the Desert Research Center, Egypt. All experimental conditions were conducted in accordance with the regulations of the Animal Welfare and Research Ethics Committee, and all protocols conformed to the International Principles for the Protection and Welfare of Animals, Faculty of Agriculture, Ain Shams University, and the Desert Research Center, Egypt. The experiment included four experimental groups, each comprising 30 male New Zealand White rabbits, aged 5 weeks (average weight 791 g ± 8.1), which were kept until they were 13 weeks. Each group contained five replicates, with six rabbits each. Each animal was placed in a cage made of galvanized wire measuring 50 × 50 × 40 cm, with unlimited feed and water provided. Also, male rabbits were selected immediately post-weaning to minimize physiological variability associated with fluctuations in reproductive hormones during female sexual maturation, which can influence metabolic activity, nutrient utilization, growth performance, and immune function. Restricting the study to males ensured a more physiologically homogeneous experimental population, thereby improving the reliability, reproducibility, and interpretability of the measured outcomes. The groups were as follows: CON: rabbits fed on a basic diet, BS: rabbits fed on a basic diet supplemented with *B. subtilis* (1.8 × 10^9^ cfu/g feed), THM: rabbits fed on a basic diet supplemented with thyme meal (10 g/kg), and CBT: rabbits fed on a basic diet supplemented with a mixture of thyme meal and *Bacillus subtilis* (5 g/kg and 1.8 × 10^9^ cfu/g, respectively). Thyme meal was received and dried for chemical analysis determination at the Desert Research Center laboratories in accordance with AOAC [33], as shown in Table 1. The nutritional requirements of rabbits were provided according to the breed requirements in accordance with the recommendations of the National Research Council (NRC, [34]) (Table 2). Additives were superimposed on the basal diet to maintain a constant macro-nutrient matrix, as the calculated nutritional dilution was negligible (<0.1%). The experiment was carried out in May and June 2024 during the summer season with the provision of clean water and pelleted feed in ad libitum at the Mariout Research Station (https://maps.app.goo.gl/JVzne9YNLf8DD1HZ9; accessed on 5 May 2024), Desert Research Center, Egypt. The lighting system at the rabbit farm was set to 16 h of light and 8 h of darkness. An integrated ventilation system combining natural and mechanical ventilation through side vents was used, allowing for natural airflow driven by wind and temperature gradients. In addition, exhaust fans were provided to ensure continuous air exchange during high temperature periods. No antibiotics or other medications were administered to the rabbits during the experimental trial to avoid interference with the effects of the dietary treatments. The experimental feed was prepared weekly and stored at room temperature in dry, airtight containers. Temperature and humidity were recorded twice daily (11:00 a.m. and 4:00 p.m.) to calculate the temperature–humidity index (THI) as described by Elbaz et al. [9]. The pulse rate (PR/min) and respiratory rate (RR/min) were determined for three rabbits from each group, according to Shebl et al. [35].

### 2.2. Growth and Carcass Traits

Growth performance was assessed by weighing rabbits individually weekly to calculate the weekly weight gain rate, expressed in g/rabbit. Feed intake was also calculated weekly by subtracting the amount of feed provided from the amount of feed remaining at the end of the week, expressed in g/rabbit. At the end of the experimental period, the feed conversion ratio (FCR, g/g) was calculated by dividing the amount of feed intake (FI, g) by the body weight gain (BWG, g). To assess carcass traits at the end of the experimental period, one rabbit from each replicate was randomly selected for slaughter by cervical dislocation (5 rabbits/group), as described by Abd El-Hamid et al. [36]. A skilled slaughterer committed to the slaughter of rabbits in experiments carried out the dissection and sacrifice of the rabbits in accordance with the World Rabbit Science Association’s guidelines [37]. Before slaughter, the rabbits were weighed after a 12 h fast. After slaughter, complete blood drainage, skinning, and evisceration, the slaughtered rabbits were weighed to determine carcass weight. Additionally, the giblets’ edible parts, including the liver, heart, spleen, kidneys, and lungs, were separated and weighed. Carcass characteristics were calculated as a percentage of the pre-slaughter weight, as described by Mohamed et al. [38].

### 2.3. Digestibility Trial and Digestive Enzyme Activity

After the experiment concluded, five rabbits were selected from each experimental group and placed separately in metabolic cages for a digestion experiment. Feces were collected, using the total collection method throughout the designated collection period, from each rabbit individually twice daily, at 10:00 a.m. and 6:00 p.m., during the five-day experimental period. The daily feed intake was recorded each morning after fecal collection. The fecal samples were ground and dried (at 65 °C for 24 h) and sent directly for analysis. To detect crude fiber (CF), crude protein (CP), ether extract (EE), and nitrogen-free extract (NFE), the feed and fecal samples from each rabbit were analyzed using the AOAC method [39] in the laboratories of the Desert Research Center (which has obtained ISO 17045). At the time of slaughter, 5 g of ileum samples were taken to measure digestive enzyme activity. Specialized commercial kits (Nanjing Jiancheng Institute of Bioengineering, Nanjing, China) were used to measure amylase, trypsin, and cellulase activity, as noted by Elbaz et al. [9].

### 2.4. Serum Parameters

At week 13, pre-slaughter blood samples were drawn from the jugular vein (centrifuged for 15 min at 3000× *g*) from 20 rabbits (5 rabbits/group) to obtain plasma for biochemical analysis at the Faculty of Agriculture, Ain Shams University laboratories. According to the procedure of Mohamed et al. [38], commercial kits were used to estimate albumin, total protein, triglycerides, high-density lipoprotein (HDL), cholesterol, and low-density lipoprotein (LDL), as well as creatinine, urea, aspartate aminotransferase (AST) and alanine aminotransferase (ALT) levels. According to the steps of Ibrahim et al. [40], thyroid function was assessed by measuring the levels of triiodothyronine (T3) and thyroxine (T4). According to the procedures of Elbaz et al. [9], commercial kits were used to determine malondialdehyde (MDA) content, glutathione peroxidase (GPx) activity, and superoxide dismutase (SOD) activity. Commercial ELISA test kits were supplied by Life Diagnostics Inc. (West Chester, PA, USA) for measuring immunoglobulin A (IgA), immunoglobulin M (IgM), and immunoglobulin G (IgG) levels.

### 2.5. Cecal Environment

During slaughter, intestinal contents were collected for microbial counts, pH, and volatile fatty acid (VFA) concentrations from five rabbits per group in the growth experiment. After collecting the cecal contents, they were placed in a sterile bag for analysis of selected microbial communities, including *Salmonella*, *Lactobacillus*, *Clostridium perfringens*, and *Escherichia coli*. The necessary dilutions of the cecal contents were carried out sequentially from 10^−1^ to 10^−8^ and placed in the appropriate agar medium for each microorganism. *Lactobacillus* was cultured in MRS agar, *E. coli* in egg yolk emulsion agar (50%), *C. perfringens* in MacConkey agar, and *Salmonella* in XLD agar. All growth conditions were maintained under appropriate environments. A pH meter (PB 10 model, Mettler Toledo, Goettingen, Germany) was used to determine the pH of the fresh cecal contents, while gas chromatography was used to measure the concentration of volatile fatty acids (VFAs) in the cecal contents (kept at −80 °C), including acetate, butyrate, and propionate.

### 2.6. Genetic Analysis

The genes SGLT-1: sodium-glucose co-transporter-1, CAT-1: cationic amino acid transporter-1, MUC-2: mucin-2, IL-6: interleukin 6, and IL-10: interleukin 10 were selected for their roles in detecting intestinal integrity (inflammation) and in nutrient absorption and transport in the small intestine from five rabbits per group. Targeted gene expression assays were performed in the Genetics Laboratory at the Faculty of Veterinary Medicine, Mansoura University. Total RNA was extracted from the small intestine mucosa (frozen in liquid nitrogen before being stored at −80 °C) using TRIZOL reagent according to the manufacturer’s protocol (Invitrogen, Carlsbad, CA, USA). Additionally, gel electrophoresis was used to verify RNA integrity. A Nanodrop ND-1000 spectrophotometer (Thermo Fisher Scientific, Waltham, MA, USA) was used to quantify RNA concentration and purity. Following the manufacturer’s instructions (Takara Biotechnology, Dalian, China) for the production of complementary DNA (cDNA), 1 microgram of total RNA was used. The primer sequences of the target genes are listed in Table 3. Under standard thermal cycling conditions, PCR reactions were performed in a real-time thermal cycling apparatus (Bio-Rad, Hercules, CA, USA). The relative expression of the target genes’ mRNA was assessed (Table 3) using GAPDH (F: TGTTTGTGATGGGCGTGAA and R: CCTCCACAATGCCGAAGT) as an internal standard (control group) via the method described by Livak and Schmittgen [41] (2^−ΔΔCt^ method).

### 2.7. Statistical Analysis

The experimental data were analyzed by ANOVA according to a completely randomized design (CRD), using the general linear model procedures in SPSS software (version 19.0). After the F-test, the significance of differences between means was assessed using Tukey’s multiple comparisons test. Before the analysis began, the homogeneity and normality of the experimental groups were assessed using Shapiro–Wilk tests. These differences were considered statistically significant at a *p*-value of 0.05.

## 3. Results

### 3.1. Welfare, Growth, and Carcass Traits

**Figure 1:**
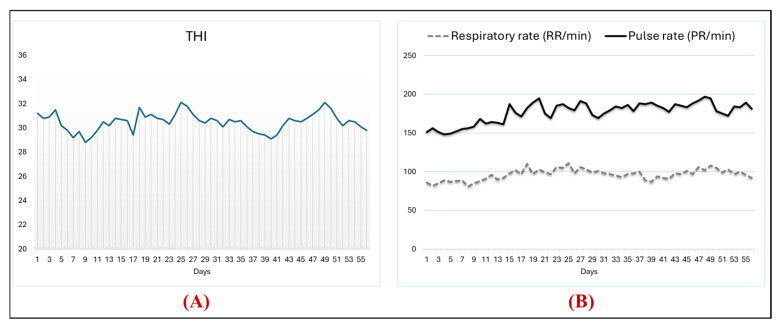
Temperature–humidity index (THI, (**A**)) values, pulse rate (PR, (**B**)), and respiratory rate (RR, **B**) during the experimental period.

Figure 1 shows the heat stress index, including the temperature–humidity index (THI) values, pulse rate (PR), and respiratory rate (RR) during the experimental period. THI values ranged from 28.8 to 32.1, RR from 148 to 197 min, and RR from 81 to 106 min, as shown in Figure 1A,C. Table 4 shows the effect of adding thyme meal and *B. subtilis* and their mixture on growth performance and carcass characteristics in rabbits under heat stress conditions. At the end of the experimental period, the experimental supplements enhanced growth performance, with increased BWG and FBW and decreased FCR compared to the control group, while FI remained unchanged. However, BWG and FCR were better in the CBT-fed rabbits compared to the other experimental groups. Additionally, compared to the control group, carcass weight and spleen were increased in the rabbits fed BS, THM, and CBT. Moreover, the remaining carcass parts, including the liver, kidneys, lungs, and heart, were unaffected by the addition of the experimental supplements to the diet of rabbits exposed to heat stress.

### 3.2. Digestive System Performance

Table 5 shows the effect of thyme meal and *B. subtilis* supplementation and their mixture on nutrient digestibility and digestive enzyme activity in rabbits under heat stress conditions. DM, CP, and CF digestibility were increased in groups fed BS, THM, and CBT compared to the control group. Moreover, DM digestibility was best digested in rabbits receiving CBT, while CP digestibility was best digested in rabbits receiving THM and CBT. Nevertheless, EE and NFE digestibility were not affected by the experimental treatments. Although digestive enzyme activity (amylase and cellulase) was unaffected by the experimental groups, trypsin levels increased in groups receiving BS, THM, and CBT compared to the control group, as shown in Table 5.

### 3.3. Serum Biochemistry

**Figure 2:**
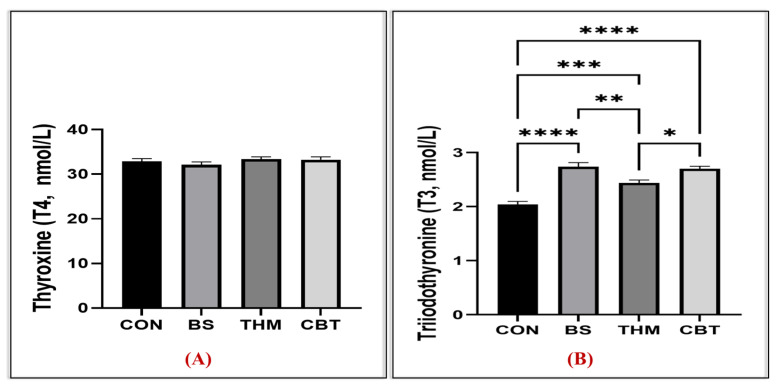
Impact of adding thyme meal, *B. subtilis*, and their blend on heat-stressed rabbits’ thyroid gland (thyroxine (T4, (**A**)) and triiodothyronine (T3, (**B**))); CON, group was fed a basal diet; BS, group was fed a basal diet with *B. subtilis*; THM, group was fed a basal diet with thyme meal; CBT, group was fed a basal diet with *B. subtilis*–thyme meal mixture. Statistical significance is denoted as follows: *p* < 0.05 (*), *p* < 0.01 (**), and *p* < 0.001 (***). Asterisks indicate levels of statistical significance: * *p* < 0.05, ** *p* < 0.01, *** *p* < 0.001, and **** *p* < 0.0001. The greater the number of asterisks, the higher the level of statistical significance.

Table 6 illustrates the effect of thyme meal and *B. subtilis* supplementation and their mixture on the lipid profile and liver and kidney function in rabbits under heat stress conditions. Cholesterol and triglyceride levels decreased in rabbits fed BS, THM, and CBT compared to the control group; however, triglyceride levels were lowest in rabbits receiving CBT and BS supplementation. Nevertheless, LDL levels tended to be lower in the CBT, THM, and BS groups compared to the control group. HDL levels were also higher in the CBT, THM, and BS groups compared to the control group. Total protein levels increased in the CBT, THM, and BS groups compared to the control group. In addition, albumin levels tended to be higher in the CBT, THM, and BS groups compared to the control group. Additionally, AST, creatinine, and urea levels reduced in the CBT, THM, and BS groups compared to the control group; however, ALT levels were not affected. As well, creatinine and urea levels were lowest in rabbits receiving CBT and BS supplementation. Moreover, triiodothyronine (T3) levels were reduced in the CBT, THM, and BS groups compared to the control group; however, thyroxine (T4) levels were not affected, as shown in Figure 2A,B.

### 3.4. Immunological and Oxidative Status

**Figure 3:**
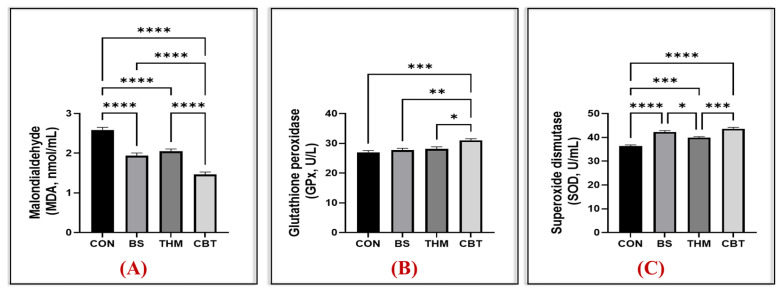
Impact of adding thyme meal, *B. subtilis*, and their blend on heat-stressed rabbits’ oxidative stability (malondialdehyde (MDA, (**A**), glutathione peroxidase (GPx, (**B**), and superoxide dismutase (SOD, (**C**)); CON, group was fed a basal diet; BS, group was fed a basal diet with *B. subtilis*; THM, group was fed a basal diet with thyme meal; CBT, group was fed a basal diet with *B. subtilis*–thyme meal mixture. Statistical significance is denoted as follows: *p* < 0.05 (*), *p* < 0.01 (**), and *p* < 0.001 (***). Asterisks indicate levels of statistical significance: * *p* < 0.05, ** *p* < 0.01, *** *p* < 0.001, and **** *p* < 0.0001. The greater the number of asterisks, the higher the level of statistical significance.

**Figure 4:**
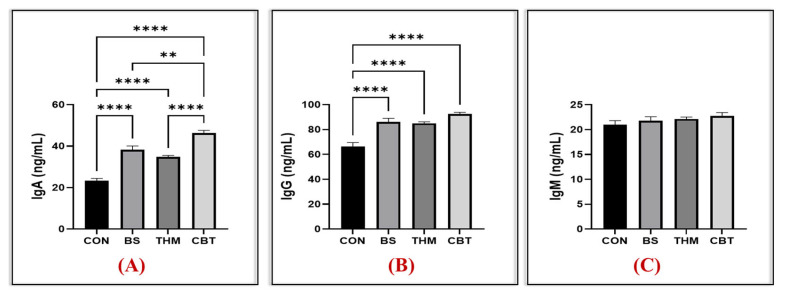
Impact of adding thyme meal, *B. subtilis*, and their blend on heat-stressed rabbits’ immune response (IgA (**A**), IgG (**B**), and IgM (**C**)); CON, group was fed a basal diet; BS, group was fed a basal diet with *B. subtilis*; THM, group was fed a basal diet with thyme meal; CBT, group was fed a basal diet with *B. subtilis*–thyme meal mixture. Statistical significance is denoted as follows: *p* < 0.01 (**). Asterisks indicate levels of statistical significance: ** *p* < 0.01, and **** *p* < 0.0001. The greater the number of asterisks, the higher the level of statistical significance.

Figure 3A–C and Figure 4A–C show the effect of thyme meal and *B. subtilis* supplementation and their mixture on oxidative stability and immune response. Compared to the control group, IgA and IgG levels increased in the groups that received the CBT, THM, and BS supplements; however, IgM levels were unaffected, as shown in Figure 3A–C. Additionally, SOD enzyme activity increased and MDA content decreased in the groups receiving CBT, THM, and BS supplementation compared to the control group. Moreover, GPx enzyme activity increased in the groups receiving CBT supplementation compared to the control, THM, and BS groups, as shown in Figure 4.

### 3.5. Cecal Microbial Count and VFAs

Table 7 shows the impact of thyme meal and *B. subtilis* supplementation and their mixture on cecal microbial count, pH value, and VFAs. *Lactobacillus* counts increased in rabbits receiving CBT, THM, and BS supplements compared to the control group, with the highest counts in the CBT group. *C. perfringens* and *E. coli* counts decreased in groups receiving CBT, THM, and BS supplements compared to the control group, with the lowest *C. perfringens* counts in the CBT and BS group. *Salmonella* counts decreased in groups receiving CBT and BS supplements compared to the control and THM groups; however, counts tended to be lower in the THM group than in the control group. The pH value tended to be lower in the groups that received CBT, THM, and BS supplements compared to the control group. The propionate concentration was higher in the groups that received CBT, THM, and BS supplements compared to the control group. Additionally, the acetate concentration was higher in the groups that received CBT and BS supplements compared to the control and THM groups, while it tended to be higher in the THM group compared to the control group. Moreover, the butyrate concentration was higher in rabbits receiving CBT compared to the control group, while it tended to be higher in groups receiving BS and THM compared to the control group.

### 3.6. Gene Expression Analysis

**Figure 5:**
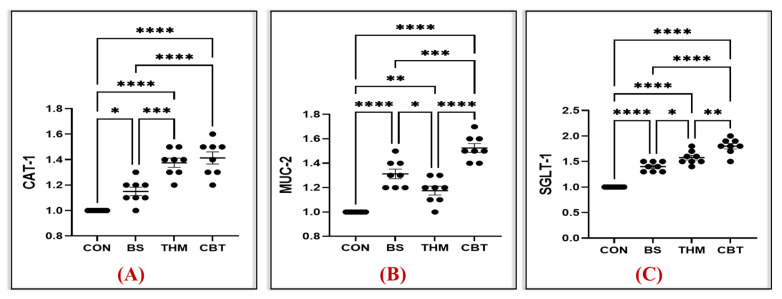
Impact of adding thyme meal, *B. subtilis*, and their blend on mRNA relative expressions of inflammation-related genes, including CAT-1 (**A**), MUC-2 (**B**), and SGLT-1 (**C**), on heat-stressed rabbits; CON, group was fed a basal diet; BS, group was fed a basal diet with *B. subtilis*; THM, group was fed a basal diet with thyme meal; CBT, group was fed a basal diet with *B. subtilis*–thyme meal mixture. Statistical significance is denoted as follows: *p* < 0.05 (*), *p* < 0.01 (**), and *p* < 0.001 (***) and *p* < 0.0001 (****).

**Figure 6:**
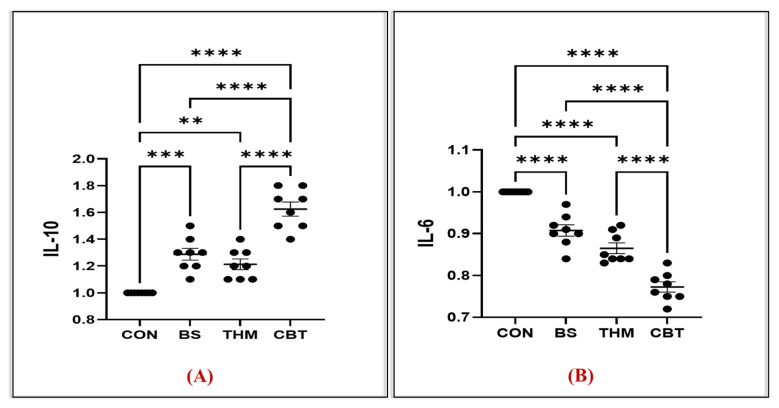
Impact of adding thyme meal, *B. subtilis*, and their blend on mRNA relative expressions of nutrient absorption and transport-related genes, including IL-6 (**A**) and IL-10 (**B**), on heat-stressed rabbits; CON, group was fed a basal diet; BS, group was fed a basal diet with *B. subtilis*; THM, group was fed a basal diet with thyme meal; CBT, group was fed a basal diet with *B. subtilis*–thyme meal mixture. Statistical significance is denoted as follows; *p* < 0.01 (**), and *p* < 0.001 (***). Asterisks indicate levels of statistical significance: ** *p* < 0.01, *** *p* < 0.001, and **** *p* < 0.0001. The greater the number of asterisks, the higher the level of statistical significance.

The effect of thyme meal and *B. subtilis* supplementation and their mixture on gene expression associated with intestinal integrity and function in rabbits under heat stress conditions is shown in Figure 5 and Figure 6. mRNA relative expressions of inflammation-related genes, including IL-6 and IL-10, are shown in Figure 6A,B. Compared with the control, CBT, THM, and BS supplements significantly increased mRNA relative expression of the IL-10 gene while decreasing mRNA relative expression of the IL-6 gene in intestinal tissue (*p* < 0.05). mRNA relative expressions of nutrient absorption and transport-related genes, including MUC-2, CAT-1, and SGLT-1, are shown in Figure 5A–C. Feeding rabbits CBT, THM, and BS supplements significantly increased mRNA relative expression of CAT-1, MUC-2, and SGLT-1 genes compared with the control group. Moreover, the highest mRNA relative expression of the CAT-1 gene was in rabbits that received CBT and THM supplements, while the highest expression of the MUC-2 and SGLT-1 genes was in rabbits that received CBT supplements.

## 4. Discussion

Climate change is a major factor affecting the expansion of rabbit farming in developing countries; despite advancements in rabbit house design, breeders still face some problems, which endanger the sustainability of rabbit breeding. Therefore, there is growing interest in using nutritional supplements as an effective strategy to mitigate the effects of heat stress and improve rabbit health and performance [9,15,16]. Among the experimental additives that have proven effective as anti-stress agents are probiotics, medicinal and aromatic plants, and their derivatives. Our study results showed that the experimental rabbits experienced heat stress during the experimental period, resulting in impaired growth and physiological performance, as well as decreased well-being. This was evidenced by increased respiratory rate, pulse rate, and THI values, reflecting the rabbits’ sensitivity to high temperatures, which decreased productivity and increased the mortality rate. A THI (temperature stress index) of 29.0 to 30.0 indicates that rabbits are experiencing severe heat stress, according to Elbaz et al. [9]. These results may be attributed to impaired physiological, digestive, and absorption functions resulting from oxidative stress and intestinal damage during heat stress [5,42,43]. Interestingly, the addition of the *B. subtilis*–thymol meal mixture enhanced growth performance, physiological status, and intestinal integrity in rabbits under heat stress conditions.

The results showed that the growing rabbits in the blend of thyme meal and *B. subtilis* treatment group achieved a higher BWG and better FCR than the rabbits in the control group under heat stress conditions. Several previous studies have reported the enhancing effects of adding thyme meal or *B. subtilis* on the growth performance of rabbits and chickens [30,44]. This suggests the role of thyme meal and *B. subtilis* supplementation in mitigating the harmful impacts of heat stress on growing rabbits through their numerous properties, including antioxidant, antimicrobial, and anti-inflammatory effects [45]. Furthermore, their growth-promoting effects are achieved by stimulating the secretion of digestive enzymes [20,30], promoting intestinal growth and maintaining its integrity [15,30,46], and inhibiting pathogenic microbes through multiple pathways (competitive exclusion and changing metabolite production) [9,16,35], thus improving nutrient utilization and growth performance.

Carcass characteristics are critical to achieving economic returns in rabbit farms. This study revealed that combining thyme meal and *B. subtilis* had a carcass weight-enhancing effect. In line with these results, Ismail et al. [47] and Elbaz et al. [9] reported that adding thyme meal or *B. subtilis* to rabbit or chicken feed increased carcass weight. This improvement in carcass weight may be attributed to the synergistic role of thyme meal and *B. subtilis* supplements in enhancing nutrient absorption, feed efficiency, and overall health through several mechanisms [47,48]. Thyme meal supplements, through their bioactive compounds, particularly thymol and carvacrol, stimulate the secretion of digestive enzymes (such as proteases, amylases, and lipases) and modify the gut microbiota [49]. They also exert antioxidant and anti-inflammatory effects, improving nutrient digestion and absorption [42,50]. This leads to increased amino acid availability for muscle growth while protecting muscle cells from oxidative stress [44,51] during heat stress. Meanwhile, *B. subtilis* improves intestinal health and structure by suppressing harmful bacteria, enhancing epithelial integrity [30,52], and producing certain enzymes and antimicrobial substances (bacteriocins and organic acids) [53,54]. This, in turn, increases nutrient availability and contributes to higher carcass weight.

The results indicated a decline in digestive function in rabbits during heat stress, characterized by reduced nutrient digestibility and decreased enzyme secretion, a finding corroborated by several previous reports [7,55]. The negative effect of heat stress is due to a decrease in the production of digestive enzymes, resulting from elevated levels of reactive oxygen species, which increase the peroxidation of fats in the cell walls of the pancreas and intestines [56]. Conversely, the addition of the *B. subtilis*–thymol meal mixture enhanced digestive function, increasing the digestibility of dry matter, protein, and fiber and boosting trypsin secretion in heat-stressed rabbits. Similarly, several studies have indicated that adding aromatic plants and their products, or probiotics, enhances the digestion of crude fiber and protein, in addition to stimulating the secretion of digestive enzymes [26,51]. Probiotics can play a role in enhancing nutrient digestion by stimulating the production of certain enzymes in the intestinal lumen [54], as well as by activating endogenous digestive enzymes through several mechanisms. These mechanisms include modifying the intestinal microbial content and structure (increasing villus length) [38], increasing the production of short-chain fatty acids during fermentation, stimulating the secretion of pancreatic enzymes (cholecystokinin and CCK), and increasing the surface area for absorption [30,46], thus enhancing nutrient digestion. Furthermore, medicinal and aromatic supplements, including thymol meal, enhance nutrient digestion through the effects of their bioactive components [9,20,44]. These components stimulate digestive glands, increase bile acid secretion, and modify the intestinal microbiota [57]. Moreover, they protect intestinal and pancreatic cells through their antioxidant effects [18,28], thereby stimulating digestive enzyme secretion and improving digestive efficiency. From the above, the *B. subtilis*–thymol meal mixtures can improve feed utilization through their synergistic effect in stimulating digestive enzyme secretion and increasing digestive efficiency.

The results indicate that the combination of thymol meal and *B. subtilis* improved the blood lipid profile and liver and kidney functions, increasing blood HDL, total protein, and albumin levels while decreasing triglycerides, cholesterol, LDL, AST, creatinine, and urea levels. Additionally, the data show a decrease in AST, creatinine, and urea, which are indicators of kidney and liver health. The enhancement of liver and kidney function in rabbits fed the mixture can be attributed to the protection of liver and kidney cells from oxidative stress damage caused by heat stress, through the proven antioxidant and anti-inflammatory properties of thymol [44,58]. These properties inhibit protein breakdown and increase the production of antioxidant enzymes, thus protecting cells from oxidative damage and preventing the leakage of intracellular enzymes (like AST) [9]. This suggests that adding the mixture to the feed of heat-stressed rabbits can increase the liver’s ability to synthesize protein [9,59], thus maintaining the body’s amino acid balance. Additionally, the mixture improves fat metabolism through several mechanisms, including inhibiting the activity of 3-hydroxy-3-methylglutaryl-CoA and cholesterol 7-alpha-hydroxylase reductase (enzymes involved in cholesterol synthesis), thus reducing cholesterol levels in the blood. Furthermore, a study reported that probiotic bacteria produce bile salt hydrolase enzyme [59,60], which has an indirect effect on lowering cholesterol levels (hypocholesterolemic). The biologically active compounds in thyme also possess cholesterol-lowering properties [18,61], in addition to anti-inflammatory and antioxidant properties. Furthermore, the experimental supplementation enhanced thyroid function, reducing metabolic disturbances in carbohydrates, fats, and minerals in heat-stressed rabbits and consequently increasing blood protein synthesis, including total blood protein, albumin, and globulin, in this study. These results demonstrate the effective role of a combination of thymol meal and *B. subtilis* in enhancing lipid metabolism and liver and kidney function in rabbits during heat stress, through its antioxidant and gut microbiome-modifying properties [30,51,60].

Among the many negative influences of heat stress is a decrease in triiodothyronine (T3) and thyroxine (T4) hormones resulting from impaired thyroid function [8], which reduces the production of these hormones, contributing to a decline in general health and a decrease in metabolic rate and growth [5], which is consistent with our findings. Despite that, adding the *B. subtilis*–thymol meal mixture led to enhanced thyroid function, as evidenced by increased T3 levels in the blood, in the current study. Similarly, several previous studies have indicated the positive effects of supplementing probiotics or aromatic plants and their products on promoting glandular function [9,16], such as increasing thyroid activity and thus boosting hormone production. The positive role of both supplementation with probiotics and thymol may be attributed to their properties that promote gut health and protect cell integrity [36,62], including thyroid gland tissue, by reducing oxidative and inflammatory damage [51], thus supporting metabolic functions and providing nutrients, thereby enhancing growth performance during heat stress.

The most deleterious effect of heat stress is oxidative stress [42], which causes oxidative damage to various organs as well as disruption of various physiological functions. The liver and intestines are among the most sensitive organs to oxidative stress. Oxidative stress is a disruption in the body’s oxidative system [49], characterized by an increase in free radicals and a decrease in antioxidant defenses [55]. The first line of defense against oxidative stress is antioxidant enzymes [51], which reduce free radicals to minimize the resulting damage. In the current study, rabbits exposed to stress exhibited oxidative stress, as evidenced by the increased MDA content and decreased GPx and SOD levels; however, feeding the rabbits a mixture of thymol meal and *B. subtilis* mitigated the effects of oxidative stress by promoting increased antioxidant enzymes, including increased GPx and SOD production, along with a significant decrease in blood MDA levels. The decrease in MDA levels indicates a marked improvement in oxidative stability in heat-stressed rabbits, especially since MDA is an indicator of cellular stress [63]. Similarly, previous studies have shown improved physiological performance, including oxidative status, in rabbits fed probiotics and aromatic plant products, through increased levels of antioxidant enzymes [60,61]. The marked improvement in oxidative status in rabbits fed the mixture is attributed to the bioactive compounds in thymol meal, which exhibit antioxidant effects by free radical scavenging and neutralizing reactive oxygen species (ROS) [58]. Simultaneously, *B. subtilis* reduces reactive oxygen species and plays a role in modifying the gut microbiota by producing several antimicrobial compounds (SCFA and polysaccharides) [15,46]. This limits pathogenic microbes’ growth, promoting gut health and nutrient absorption [8,30], and thus enhancing oxidative stability in heat-stressed rabbits. Therefore, adding a combination of *B. subtilis* and thyme meal may enhance rabbit health by protecting against heat stress through safeguarding cells from oxidative stress in growing rabbits.

Heat stress significantly weakens the immune response of rabbits by reducing antibody production, decreasing immune cell function [9], and lowering protein synthesis in the immune organs and lymphoid tissue, and damaging the intestinal lining, leading to immunosuppression [42] and decreasing the weight of immune organs (spleen and thymus), and thus making rabbits more susceptible to infection and deteriorating health. Numerous scientific studies have shown that dietary interventions by means of supplements enhance immune functions in rabbits exposed to heat stress [9,16], which is consistent with our findings. Immunoglobulins are essential components of the rabbit’s adaptive immune system during stress, providing protection against pathogens [64]. They recognize and bind to specific antigens, thus mitigating the negative effects of stress. Combined *B. subtilis* and thyme meal enhanced the immune response, which is reflected in an increase in IgA and IgG and an increase in the relative weight of the spleen. Consistent with our immunological data, numerous studies have demonstrated the role of *B. subtilis* and thymol supplements in boosting immune organ and immunoglobulins [45,53,65], thereby enhancing the immune status of rabbits. In addition, atrophy of the lymphatic organs (spleen) is a symptom of heat stress [66,67], which is consistent with our findings. However, the current study showed that the spleen growth index in rabbits increased significantly when a mixture of *B. subtilis* and thymol meal was added during heat stress. Similarly, a study noted that the addition of *B. subtilis* significantly increased the spleen and thymus index in rabbits exposed to heat stress [30,65]. These results indicate an improvement in the overall immune status of heat-stressed rabbits through increased antibody production and lymphatic organs index via adding the *B. subtilis*–thymol meal mixture.

Rabbits exposed to heat stress will suffer from weakened immunity and be more susceptible to a variety of diseases [8], including diarrhea and enteritis, which is due to an imbalance of intestinal bacteria that alters the mechanism of fermentation and digestion in rabbits [68], making them more prone to disease. This imbalance in gut bacteria is caused by elevated levels of reactive oxygen species [69], leading to an overgrowth of pathogenic bacteria (*C. perfringens* and *E. coli*) and a decrease in beneficial microbes [55], thus compromising gut health. However, the *B. subtilis*–thymol meal mixture showed antimicrobial activity, as it increased the number of *Lactobacillus* while decreasing the number of *C. perfringens*, *E. coli*, and *Salmonella*. Numerous studies have demonstrated that *B. subtilis* has acquired antimicrobial properties against multiple pathogenic bacteria through several mechanisms, including competitive action and the production of antimicrobials (VFA) [30,70], as well as consuming oxygen in the intestine [30], inhibiting the growth of pathogenic anaerobic bacteria (*E. coli*). Similarly, a study suggests that adding thyme to rabbit feed treats diarrhea and bacterial enteritis, due to its potent antimicrobial properties [51,62], through biologically active compounds, which reduced the numbers of *C. perfringens* and *E. coli* in both the small and large intestines. Combining *B. subtilis* and thymol meal may represent a competitive approach to combating infections with enteric pathogens in heat-stressed rabbits.

Disruption of the gut microbiome resulting from heat stress leads to large changes in fermentation processes [55], altering the concentrations and production of VFAs, which in turn causes intestinal damage and inflammation [71]. VFAs are metabolic products resulting from the fermentation of dietary fiber by bacteria in the rabbit’s intestines [72]. They play an important role in regulating intestinal health and as a basic material for energy production [73], as well as in reducing intestinal acidity, which inhibits the invasion and colonization of pathogens [74], thus improving feed utilization efficiency and performance. Acetate helps build a balanced microbiological environment in the gut by regulating the competition between *Bifidobacteria* and intestinal pathogens [75]. In addition, butyrate had a positive role in maintaining a modified gut microbiome by inhibiting the colonization of *C. perfringens* and *Salmonella*, which are causes of necrotizing enteritis, in the intestines [76]. VFAs also play a crucial role in regulating the gut microbiome barrier by promoting the secretion of antimicrobial peptides and mucus production in the gut [77], which is attributed to increased mucus gene expression and the induction of apoptosis [72]. In this study, adding the *B. subtilis*–thymol meal mixture affected the concentrations of VFAs in the cecum of heat-stressed rabbits. Rabbits’ cecum fed the *B. subtilis*–thymol meal mixture contained the highest VFA levels, including acetate and propionic and butyric acids. Based on this, a study showed that adding probiotics to the diet of weaned rabbits stimulates the production of VFAs [78]. Similarly, weaned pigs showed an increase in VFA concentration as a result of thymol feeding [79]. The marked increase in the concentration of VFAs in the cecum of rabbits that consume the mixture may be attributed to an increase in the number and activity of *Lactobacillus* bacteria in the cecum [73], which could contribute to improving nutrient digestibility. Additionally, the increased production of short-chain fatty acids may be attributed to the dietary fiber in thymol meal. Several reports have demonstrated that fibers containing beta-glucans, pectin, and arabinoxylans have an effect that promotes short-chain fatty acid production [80,81]. These results indicate that adding the *B. subtilis*–thymol meal mixture increases the concentration of VFAs by modifying the microbiota in the cecum, which increases its productivity and fermentation activity, leading to stimulating cytokine production and repairing the intestinal mucosa, alleviating intestinal inflammation, facilitating the morphological advancement of the mucosa [72,82], and thus improving intestinal health in heat-stressed rabbits.

Rabbits exposed to heat stress suffer damage to intestinal mucosal tissue and a weakened anti-inflammatory response due to elevated levels of reactive oxygen species [5], which increase lipid peroxidation in the intestinal cell walls, leading to altered gene expressions of mucus, nutrient transporters, and markers of immunity and inflammation [83,84]. Despite that, using feed additives has led to enhanced gene expression during heat stress, particularly of genes associated with gut health [84] and oxidative-immune status [85]. Therefore, we measured the expression level of gut integrity and amino acid transporter genes. In the current study, the expression of the CAT-1, MUC-2, SGLT-1, and IL-10 genes increased, while the expression of the IL-6 gene decreased, in rabbits fed a mixture of thyme meal and *B. subtilis.* Similarly, previous studies have shown that adding probiotics to rabbit feed increased the expression of the SGLT-1, CAT-1, and MUC-2 genes [36,86,87], thereby improving nutrient absorption. Additionally, the concentrations of IL-10 and SGLT1 in poultry fed thyme were increased, while IL-6 was significantly decreased [88,89]. The role of probiotics in enhancing gene expression may stem from the effective modification of the gut microbiota. This has been shown to contribute effectively to reducing pathogen colonization, strengthening the epithelial barrier, regulating cytokine secretion [36,90], preserving the cell junctional architecture of CLDN-1, occludin [91], and developing the intestinal immune system, thereby increasing barrier function and the upregulation of gene expression [86]. In addition, thymol (one of its main active components) in thyme meal possesses antibacterial, antioxidant, immunomodulatory, and anti-inflammatory properties [89,92]. These properties act as a protective factor for the body’s systems and organs (like the intestines and liver) [93,94] by reducing free oxygen and protecting cells from the damaging effects of oxidative stress, thereby increasing gene expression associated with intestinal and liver health. Therefore, combining thymol meal and *B. subtilis* may have a synergistic effect, mitigating intestinal damage caused by heat stress and increasing the expression of the CAT-1, MUC-2, IL-10, and SGLT-1 genes in the intestine, thus enhancing intestinal integrity and nutrient transport in the intestine.

## 5. Conclusions

Our study shows that adding the thymol meal–*B. subtilis* mixture to the feed of heat-stressed weaned rabbits effectively improved their productivity and health. The addition of the mixture enhanced rabbit health by improving the antioxidant status, immune markers, and liver and kidney function. Furthermore, it enhanced growth performance in heat-stressed rabbits by increasing nutrient digestibility and digestive enzyme production, as well as increasing the expression of the CAT-1 and SGLT-1, as well as improving intestinal integrity. Additionally, the mixture increased VFA levels in the cecum, reduced harmful microbes, and upregulated the expression of the MUC-2 and IL-10 genes, thus promoting intestinal integrity in rabbits under heat stress conditions. Therefore, it can reduce the deterioration associated with heat stress by supporting a nutritional strategy based on the thymol meal and *B. subtilis* mixture, which directly contributes to maintaining the integrity of the intestines, liver, kidneys, and physiology, thus promoting the health, growth, and performance of rabbits.

## Figures and Tables

**Table 1:** Chemical composition of thyme meal.

Ingredients	%
Dry matter	91.4
Crude protein	11.6
Crude fiber	8.7
Crude fat	3.1

**Table 2:** The basal diet (control), components, and chemical composition.

Components	%
Alfalfa hay	27.1
Yellow corn	14.0
Soybean meal (44%)	15.0
Barley	17.0
Wheat bran	19.0
Sunflower meal	2.00
Limestone	1.00
Dicalcium phosphate	2.15
Premix (Vit-Min) *	0.30
DL-methionine	0.15
NaCl	0.30
Molasses	2.00
**Chemical composition**	
Dry matter	91.4
Crude protein	17.0
Energy (kcal/kg)	2585
Crude fiber	11.3
Crude fat	3.15
Calcium	1.14
Total phosphorus	0.71
Methionine	0.58
Lysine	0.97

* Supplied per 1 kg diet: 6000 IU vit. A; 2.0 mg vit. K3; 2.0 mg vit. B6; 900 IU vit. D3; 2.0 mg vit. B1; 10.0 mg vit. B5; 40 mg vit. E; 4.0 mg vit. B2; 0.010 mg vit. B12; 5.0 mg vit. PP; 50.0 mg Fe; 0.05 mg B8; 3.0 mg B9; 250 mg choline; 8.5 mg Mn; 0.20 mg I; 5.0 mg Cu; 50.0 mg Zn; and 0.00 mg Se.

**Table 3:** Primer sequences for quantitative real-time PCR.

Gene	Accession No.	Primer Sequences
CAT-1	XM_002721425.3	F: CCAGTCTATTAGGTTCCATGTTCC R: CGATTATTGGCGTTTTGGTC
SGLT-1	NM_001101692.1	F: GATTTCCCGTATGATTACCGAG R: AAGAGGGAGACAACCACAACG
MUC-2	U85787.1	F: TATACCGCAAGCAGCCAGGT R: GCAAGCAGGACACAGACCAG
IL-10	NM001082045.1	F: AAAAGCTAAAAGCCCCAGGA R: CGGGAGCTGAGGTATCAGAG
IL-6	NM_001082064.2	F: ACGATCCACTTCATCCTGCG R: GGATGGTGTGTTCTGACCGT

**Table 4:** Impact of adding thyme meal, *B. subtilis*, and their blend on heat-stressed rabbits’ growth and carcass traits.

Parameter		CON	BS	THM	CBT	SEM	*p* Value
Growth index	IBW, g	790.7	791.4	789.8	790.5	2.955	0.314
	FBW, g	1803.2 ^c^	1881.5 ^b^	1897.3 ^b^	1942.6 ^a^	7.281	˂0.001
	BWG, g	1013.5 ^c^	1090.1 ^b^	1106.5 ^b^	1152.1 ^a^	3.773	˂0.001
	FI, g	5212	5184	5192	5186	11.045	0.216
	FCR, g/g	5.14 ^a^	4.76 ^b^	4.69 ^b^	4.50 ^c^	0.133	0.001
Carcass traits	LBW, g	1794 ^c^	1905 ^b^	1911 ^b^	1955 ^a^	4.261	˂0.001
	CW, %	68.71 ^b^	74.33 ^a^	73.82 ^a^	75.01 ^a^	1.585	0.001
	Liver, %	3.06	3.11	3.08	3.02	0.092	0.085
	Spleen, %	0.07 ^b^	0.10 ^a^	0.09 ^a^	0.11 ^a^	0.031	0.007
	Kidneys, %	0.69	0.68	0.70	0.69	0.004	0.091
	Lungs, %	0.71	0.72	0.71	0.70	0.012	0.102
	Heart, %	0.31	0.29	0.30	0.31	0.005	0.087

FI, feed intake; BWG, body weight gain; FCR, feed conversion ratio; LBW, live body weight; CW, carcass weight; CON, group was fed a basal diet; BS, group was fed a basal diet with *B. subtilis*; THM, group was fed a basal diet with thyme meal; CBT, group was fed a basal diet with *B. subtilis*–thyme meal mixture. Mean values followed through various superscript letters in the same row are significantly different (*p* < 0.05).

**Table 5:** Impact of adding thyme meal, *B. subtilis*, and their blend on heat-stressed rabbits’ nutrient digestibility and digestive enzyme activity.

Parameter		CON	BS	THM	CBT	SEM	*p* Value
	DM, %	64.8 ^c^	66.3 ^b^	67.0 ^b^	68.2 ^a^	2.840	0.001
Nutrient digestibility	CF, %	47.6 ^c^	49.4 ^ab^	48.5 ^b^	50.1 ^a^	1.672	0.020
	CP, %	65.7 ^c^	67.5 ^b^	69.2 ^a^	70.3 ^a^	2.211	˂0.001
	EE, %	79.3	80.2	79.8	80.7	3.065	0.133
	NFE, %	50.8	50.3	51.1	51.4	2.443	0.197
Digestive enzyme activity	TRY, KU/mg	1.84 ^c^	2.27 ^b^	2.41 ^ab^	2.73 ^a^	0.071	0.001
	AMY, U/g	3.61	3.62	3.57	3.60	0.105	0.110
	CEL, U/g	15.31	15.26	15.40	15.37	0.531	0.204

CF, crude fiber; CP, crude protein; EE, ether extract; NFE, nitrogen-free extract; CEL, cellulase; AMY, amylase; TRY, trypsin; CON, group was fed a basal diet; BS, group was fed a basal diet with *B. subtilis*; THM, group was fed a basal diet with thyme meal; CBT, group was fed a basal diet with *B. subtilis*–thyme meal mixture. Mean values followed through various superscript letters in the same row are significantly different (*p* < 0.05).

**Table 6:** Impact of adding thyme meal, *B. subtilis*, and their blend on heat-stressed rabbits’ lipid profile and liver and kidney functions.

Parameter		CON	BS	THM	CBT	SEM	*p* Value
	Triglycerides, mg/dL	182 ^a^	126 ^c^	153 ^b^	119 ^c^	2.971	0.001
Lipid profile	Cholesterol, mg/dL	223 ^a^	186 ^b^	191 ^b^	162 ^c^	4.262	0.001
	LDL, mg/dL	97.1 ^a^	88.4 ^b^	93.5 ^ab^	84.5 ^b^	3.115	0.020
	HDL, mg/dL	38.5 ^c^	43.8 ^ab^	42.2 ^b^	45.4 ^a^	2.542	0.010
Liver and kidney functions	Total protein, g/dL	4.72 ^d^	6.05 ^b^	5.41 ^c^	6.73 ^a^	0.210	˂0.001
	Albumin, g/dL	3.53 ^b^	3.71 ^a^	3.64 ^ab^	3.74 ^a^	0.305	0.018
	AST, U/L	57.4 ^a^	53.8 ^b^	50.6 ^bc^	48.3 ^c^	3.052	0.006
	ALT, U/L	41.5	40.3	41.1	39.6	2.181	0.083
	Creatinine, g/dL	0.87 ^a^	0.62 ^c^	0.73 ^b^	0.60 ^c^	0.948	0.010
	Urea, g/dL	35.8 ^a^	30.7 ^c^	32.4 ^b^	29.6 ^c^	1.061	0.001

HDL, high-density lipoprotein cholesterol; LDL, low-density lipoprotein; AST, aspartate aminotransferase; and ALT, Alanine aminotransferase; CON, group was fed a basal diet; BS, group was fed a basal diet with *B. subtilis*; THM, group was fed a basal diet with thyme meal; CBT, group was fed a basal diet with *B. subtilis*–thyme meal mixture. Means within a row with various letters (a–d) are statistically significantly different (*p* < 0.05).

**Table 7:** Impact of adding thyme meal, *B. subtilis*, and their blend on heat-stressed rabbits’ gut environment (cecal microbial count, pH value, and VFAs concentration).

Title 1		CON	BS	THM	CBT	SEM	*p* Value
	*Lactobacillus*	5.26 ^d^	6.38 ^b^	5.85 ^c^	6.77 ^a^	0.084	˂0.001
Microbial enumeration	*C. perfringens*	6.51 ^a^	5.19 ^c^	5.76 ^b^	5.08 ^c^	0.151	˂0.001
	*E. coli*	7.02 ^a^	5.16 ^bc^	5.58 ^b^	4.89 ^c^	0.067	0.001
	*Salmonella*	3.73 ^a^	2.83 ^b^	3.21 ^ab^	2.24 ^c^	0.113	0.010
VFAs concentration	pH	6.51 ^a^	6.40 ^b^	6.46 ^ab^	6.38 ^b^	0.104	0.020
	Acetate	32.7 ^b^	35.2 ^a^	34.1 ^ab^	35.8 ^a^	0.815	0.008
	Butyrate	5.43 ^b^	5.52 ^ab^	5.50 ^ab^	5.65 ^a^	0.094	0.037
	Propionate	2.16 ^b^	2.54 ^a^	2.47 ^a^	2.53 ^a^	0.007	0.001

CON, group was fed a basal diet; BS, group was fed a basal diet with *B. subtilis*; THM, group was fed a basal diet with thyme meal; CBT, group was fed a basal diet with *B. subtilis*–thyme meal mixture. Means within a row with various letters (a–d) are statistically significantly different (*p* < 0.05).

## Data Availability

The original contributions presented in this study are included in the article. Further inquiries can be directed to the corresponding authors.

## Abbreviations

The following abbreviations are used in this manuscript:

ALT	Alanine aminotransferase
AMY	Amylase
AST	Aspartate aminotransferase
BS	Rabbits received a basal diet with *B. subtilis*
BWG	Body weight gain
*C. perfringens*	*Clostridium perfringens*
CAT-1	Cationic amino acid transporter-1
CBT	Rabbits received a basal diet with *B. subtilis*-thyme meal mixture
CEL	Cellulase
CF	Crude fiber
CON	Rabbits received a basal diet
CP	Crude protein
CW	Carcass weight
E. coli	Escherichia coli
EE	Ether extract
FCR	Feed conversion ratio
FI	Feed intake
GPx	Glutathione peroxidase
HDL	High-density lipoprotein
IL-6	Interleukin 6
L-10	Interleukin 10
LDL	Low-density lipoprotein
MDA	Malondialdehyde
MUC-2	Mucin 2
NFE	Nitrogen-free extract
PR	Pulse rate
RR	Respiratory rate
SGLT-1	Sodium-glucose co-transporter-1
SOD	Superoxide dismutase
T3	Triiodothyronine
T4	Thyroxine
THI	Temperature-humidity index
THM	Rabbits received a basal diet with thyme meal
TRY	Trypsin
